# Subjective and objective stress during body exposure: a comparison of adolescents with anorexia nervosa versus high body dissatisfaction

**DOI:** 10.3389/fpsyt.2024.1452923

**Published:** 2025-01-20

**Authors:** Valeska Stonawski, Louisa Kuper, Nicolas Rohleder, Gunther H. Moll, Hannah Fischer, Anne-Christine Plank, Tanja Legenbauer, Oliver Kratz, Stefanie Horndasch

**Affiliations:** ^1^ Department of Child and Adolescent Mental Health, University Hospital Erlangen, Friedrich-Alexander University Erlangen-Nürnberg (FAU), Erlangen, Germany; ^2^ Chair of Health Psychology, Department of Psychology, Friedrich-Alexander-Universität Erlangen-Nürnberg, Erlangen, Germany; ^3^ Department for Child and Adolescent Psychiatry, Psychosomatic and Psychotherapy, LWL University Hospital of the Ruhr-University Bochum, Hamm, Germany

**Keywords:** anorexia nervosa, adolescents, body exposure, stress, cortisol, alpha-amylase

## Abstract

**Objective:**

Body dissatisfaction (BD) is a risk factor for the development of an eating disorder (ED) and a negative predictor for treatment outcome in adolescents with anorexia nervosa (AN). As a clinical core symptom and a relevant maintaining factor of AN, body image disturbance and BD are highly relevant target variables for therapeutic interventions. Body exposure (BE) was found as being effective for reducing BD in adolescents with EDs and high BD. However, the underlying mechanisms of BE are still not clear, with habituation processes being discussed as one possible mechanism.

**Methods:**

Affective and neurobiological processes during a four-session computer-based BE intervention were investigated. Within a controlled design comparing adolescents with AN (*n* = 34) vs. adolescent patients with high BD (*n* = 17) but without a diagnosed ED, subjective (stress ratings; 11-point Likert scale) and objective (salivary cortisol and alpha-amylase [sAA]) stress measures were assessed at four time points at each exposure session (start, +10 min, +30 min/end, +60 min/recovery). ED and depressive psychopathology were assessed via self-rating questionnaires.

**Results:**

A between-session habituation effect was found for subjective stress ratings and sAA levels with decreasing scores throughout the four sessions. A within-session habituation was found for cortisol levels. Higher psychopathology was associated with subjective stress ratings. There were no group differences between AN and BD regarding ED psychopathology or subjective or objective stress measures. Subjective and objective stress measures were mainly not associated with each other.

**Conclusions:**

Habituation processes were found for subjective and objective stress, which might enhance motivation to continue BE interventions and thus increase their impact. BD seems to be a transdiagnostic phenomenon with BE as a successful intervention independent of psychiatric diagnosis. Current findings should be validated in larger samples, and the hypothesis of a transdiagnostic BD should be investigated in future research.

## 1 Introduction

Body image as a multidimensional construct encompasses the perception, cognitions, emotions, and behaviors in relation to one’s own body. Body image disturbance is a core clinical feature of eating disorders (ED), especially of anorexia nervosa (AN), and presents itself thereby typically as a distorted perception so that adolescents with AN for example perceive themselves as being fat despite being objectively underweight. It is discussed as a maintaining factor of AN and as a risk factor for a negative course of AN (1). Body image disturbance often persists after recovery and has been shown as a risk factor for relapse (2, 3). Furthermore, body dissatisfaction (BD) describes dissatisfaction with one’s own whole body, certain body parts, or one’s own physical appearance and often goes along with the desire to lose weight or look more attractive. It is associated, among other things, with the frequency of comparisons with other (unrealistic or thin) bodies, e.g., on social media (4). In adolescents with AN, BD has been shown as a negative predictor of a clinically significant change of inpatient treatment outcome (5). Additionally, BD is a common phenomenon among non-clinical adolescents, especially in girls and overweight adolescents, with a highly varying prevalence between 19% and 83% (6, 7). It is associated with depressive symptoms in adolescents (8–10) and considered as a risk factor for the development of EDs (11, 12).

Knowing the importance of body image disturbance for the course of AN and the risk potential of BD in non-clinical and clinical samples, it is crucial to integrate body-related interventions in the treatment of AN (13) but also in the care of “risk samples” with high BD. Therefore, body exposure (BE) is highly recommended and has been established as a central intervention in the treatment of EDs (13–15). During BE, patients are confronted with the image of their own body—typically in several sessions and usually wearing tight clothes or underwear to view the body shape and size as clearly as possible. While BE in front of a mirror is the most common type of BE (16), there are also other variants, e.g., in virtual reality (17). Furthermore, a distinction is made between guided and pure BE: During guided BE, the body is viewed according to a fixed pattern or sequence (e.g., from head to feet or from pleasant to unpleasant body parts), and patients are guided to give a detailed, neutral description of their own physical appearance [e.g., (18)]. In contrast, pure confrontation is performed without any instructions. BE has been shown to be effective in reducing BD and body avoidance, not only in patients with AN but also in women with high BD without an AN diagnosis (16, 19, 20), with effects for both variants, the guided and pure BE (21–23).

Despite the clinical relevance and effectiveness of BE, the underlying mechanisms are still not clarified (16, 24). Besides the rationales of an attention bias modification, a reduction of body perception distortion, or a change of dysfunctional cognitions, psychological and biological habituations are discussed as possible mechanisms of BE (16, 24). Being confronted with one’s own body, individuals with EDs (mainly investigated for bulimia nervosa [BN]), and high body dissatisfied women show negative cognitive, emotional, and altered physiological responses [e.g., (25–27)]. Repeated confrontation with such stressful or unpleasant stimuli, as known from anxiety and ED research, is then accompanied by a decrease in the affective and biological stress response known as habituation (28–30); thereby, a distinction is made between habituation in a single session (within-session habituation) and habituation over several sessions (between-session habituation) (30, 31).

For the assessment of stress reactivity, subjective measures in terms of distress or emotion ratings as well as objective biomarkers of the acute stress reaction are used. Former studies investigating the subjective course of emotions found an affective habituation in terms of decreasing distress or negative emotions within a single exposure session (32) or across several sessions (21, 22, 27) in women with mixed EDs or high BD. Comparable, subjective between-session habituation for daily exposures to feared food was associated with a positive treatment outcome in adolescents with diverse EDs (30).

Regarding objective stress measures, cortisol is typically used as a correlate of an activated hypothalamic–pituitary–adrenal (HPA) axis representing the key system of the neuroendocrine stress response, with dysfunctions being associated with psychiatric disorders or a higher risk for diseases (33–35). In patients suffering from acute AN, elevated basal levels were found as well as a blunted cortisol reactivity (36–38). With proceeding weight gain, basal cortisol levels have been shown to normalize, but altered cortisol reactivity persists even within a normal weight range (36). In addition, salivary alpha amylase (sAA) is another marker of the acute stress reaction, reflecting a “faster” sympathetic nervous system [SNS; (39–41)] activity. A decreased activation of the SNS in terms of an attenuated sAA response to a stressor was found in patients with acute restrictive AN (37, 42). Interestingly, investigating reactivity towards a psychosocial stressor, Monteleone et al. reported a dissociation between the HPA axis and the SNS response in underweight individuals with AN who exhibited a strong cortisol reactivity but an almost completely lacking increase of sAA (compared with control groups) (42). The functionality of the HPA axis and the SNS has been investigated often separately from each other, although they are related systems (43, 44). While the HPA axis and the SNS have inverse circadian patterns, in response to a stressor, they both react with increasing values in order to prepare a person’s coping with the stressor (43). Hereby, according to different downstream processes, SNS reacts within minutes with, among others, rising sAA values and effects on heart rate, blood pressure, or attention, whereas cortisol values peak within 20 min or more, modulating glucose levels or immune processes (43, 45).

With regard to neurobiological stress reactivity and habituation during BE, fewer and inconclusive findings are available, with to the best of the authors’ knowledge none specifically regarding AN: In women with BN who obtained six therapeutic sessions of BE in front of a mirror, a decrease of salivary cortisol levels within the initial and the final session was observed, which could be interpreted as within-session habituation of the neuroendocrine stress response; furthermore, but only during pure not guided BE, a tendency towards a stronger habituation in the last session compared with the first one was found hinting additionally to a between-session habituation (22). Besides general higher cortisol levels in women with mixed EDs compared with healthy controls (HC), however, no within-habituation effect regarding cortisol could be found in a single 40-min BE task (32). Looking at other stress-related biomarkers and comparing subjective and objective measures revealed missing associations: Servián-Franco et al. observed a dissociation of the subjective and objective reaction to BE in young women: high compared with low body dissatisfied women reported more negative emotions and cognitions but showed a decreased physiological response in terms of skin conductance and heart rate (HR), which was hypothesized as hint to a passive-behavioral inhibited coping style (25). Comparably, evaluating three sessions of BE in patients with BN, Trentowska et al. found a cognitive-affective within- and between-session habituation, which however did not correlate with the autonomic responses in HR or skin conductance (27).

In sum, studies investigating subjective and objective stress during BE, in particular regarding AN and adolescents, are scarce or even lacking, which results in an insufficient understanding of the underlying mechanisms. Therefore, in the current study, we investigated the subjective and objective stress response to a computer-based BE in which a guided confrontation with photos of the own body was carried out according to an adapted version of a manualized body image treatment program. Thereby, adolescents with AN were compared with adolescents with high BD who were all treated in an inpatient or day-clinic setting due to their primary psychiatric diagnosis, which allowed for standardized, comparable environmental conditions and the practicability of several study appointments. A comparison of the AN group with the clinical BD group should further facilitate the analysis of similarities and differences in the effects and underlying processes of BE between these two groups that might benefit from BE based on former studies. We hypothesized a between- and a within-session habituation for the affective stress ratings as subjective and for cortisol and sAA as objective stress measures. Furthermore, we hypothesized higher levels in general and a blunted stress reactivity for both cortisol and sAA in patients with AN compared with adolescents with high BD, which manifests itself in the form of smaller habituation. Due to former inconsistent or lacking findings, in exploratory analyses, we investigated the associations between subjective and objective stress measures and treatment outcome in terms of psychopathology.

## 2 Materials and methods

### 2.1 Study design and procedure

The current data were collected within the FRAnconian Longitudinal study of Anorexia Nervosa in Adolescents (FRALANA) evaluating the effectiveness of treatment services for adolescents with AN and investigating its underlying mechanisms. In the intervention module, a standardized computer-based BE consisting of four sessions over a period of 2.5 weeks was evaluated in a controlled design: In each session, adolescents were confronted with photos of their own body on a computer screen; thereby, BE followed a guided approach according to a manualized body image treatment program (18) to promote a neutral reaction towards and description of the own body parts. Effects of the BE were evaluated, among other things, in regard to adolescents’ subjective and objective stress presented here; more information on the FRALANA intervention module can be found in the associated study protocol (46). Furthermore, in order to identify AN-specific processes using a controlled study design, adolescents with AN were compared with adolescents with high BD, who were both in inpatient or day-clinic psychiatric treatment. The BD group was surveyed at two locations: *n* = 3 adolescents of the BD group (18%) were recruited in a second study center. All adolescents and their parents, respectively, gave informed consent before participation. Ethical approval for the study was granted by the local ethics committee of the Medical Faculty. The study was conducted in accordance with the Declaration of Helsinki.

All adolescents participated in four exposure sessions (T1–T4) framed by a pre- and a post-session; all sessions took place within 2.5 weeks, with two exposure sessions per week and at least 2 days in between. Each session was standardized: The pre-session consisted of psychodiagnostics (see below), a psychoeducation regarding exposure interventions, and an explanation of the exposure sessions, followed by taking standardized photos from frontal and lateral views in standardized tight-fitting sportswear (black sports bra and tight shorts). At the post-session, psychodiagnostics were repeated and all participants received an expense allowance in terms of 20€ for their participation. During the four intervention sessions, which all took place at a comparable time in the early afternoon (*p* = .328–.891; starting times: AN: *M_T1–T4_* = 1:36–1:47 pm, *SD_T1–T4_* = 0:50–1:00; BD: *M_T1–T4_* = 1:21–1:53 pm, *SD_T1–T4_* = 0:41–1:04), the participants were confronted with the photos of themselves on a computer screen. Here, they were guided through the sessions by an audio file playing back standardized instructions according to the treatment manual of Vocks et al., in which participants are guided to look at 12 different parts of their body (18). To assess the stress response during and after the body exposure, stress ratings were assessed and saliva samples were collected at four time points: The first saliva sample was taken before BE (t1; 0′), the second 10 min after the start of BE (t2; +10′/min), the third at the end of BE (t3; +30′), and the last 30 min after the end of each session (t4; +60′) as a recovery sample.

### 2.2 Sample

Female adolescents between the ages of 10 and below 18 years who were all treated in an inpatient or day-clinic psychiatric treatment setting as patients due to their primary psychiatric diagnosis were included in the current study. For the AN group, participants had to be diagnosed with AN (typical or atypical) according to the ICD-10 criteria by an experienced child psychiatrist or psychologist; from admission to the start of the study intervention, all adolescents were required to have gained weight up to a body weight >10th body mass index (BMI) age percentile. This corresponded to a weight above the underweight range to avoid habituation at very low weight in participants with AN as recommended elsewhere [see details, e.g., in (14, 16)]. For the BD group, adolescents had to be diagnosed with a psychiatric disorder other than an ED and be highly body dissatisfied according to the EDI-2 subscale “body dissatisfaction” [highest quartile: >75th percentile] and clinical judgment of an experienced child psychiatrist or psychologist, and their weight had to be within the normal range (BMI >10th age-percentile). Antidepressant or antipsychotic medication was allowed; however, acute psychotic symptoms, use of illegal substances, medication with sedating effects, chronic somatic diseases, intellectual disability (IQ < 85), and insufficient understanding of the German language were exclusion criteria.

A total of 51 female adolescents between the ages of 11.6 and 17.8 years were included in the current analyses, who attended all (*n* = 47) or at least three exposure sessions (*n* = 4). *N* = 4 adolescents were not included due to attendance of only two or less exposure sessions; one of these adolescents cancelled actively her participation due to her high emotional involvement, and the other three were discharged from treatment before study termination. Within the AN group including *n* = 34 adolescents (74% in inpatient setting), the majority had an ICD-10 diagnosis of a typical AN (restrictive type: *n* = 20; binge/purge type: *n* = 10) and *n* = 4 the diagnosis of an atypical AN (F50.1). Comorbid diagnoses in the AN group were a depressive episode (*n* = 13), anxiety disorders (social phobia: *n* = 6; GAD: *n* = 4), a posttraumatic stress disorder (PTSD), or an obsessive-compulsive disorder (OCD; each *n* = 2). Within the BD group covering *n* = 17 adolescents with high BD (29% in an inpatient setting), all adolescents had a depressive episode as main (*n* = 14) or comorbid diagnosis (*n* = 3); other main diagnoses were an anxiety disorder (*n* = 2) and an OCD (*n* = 1). Other comorbidities were anxiety disorders (social phobia: *n* = 4; generalized anxiety disorder, GAD: *n* = 3; panic disorder: *n* = 1), PTSD (*n* = 4), and a borderline personality disorder or a trichotillomania (each *n* = 1). Overall, *n* = 29 adolescents (AN: *n* = 18, BD: *n* = 11) had more than one psychiatric diagnosis; both groups did not differ in the ratio of comorbid diagnoses (see **Table 1**). Until the start of BE, the average treatment duration within the AN group was 16.4 weeks with an average weight gain of +7.7 kg (mean BMI at start of BE: 18.0 kg/m^2^); within the BD group, the average treatment duration was 7.8 weeks with an average weight gain of +1.0 kg (mean BMI at start of BE: 24.2 kg/m^2^). Due to missing data in one complete exposure session, in single saliva samples or single data points after laboratory analyses as well as due to exclusions after quality control, sample sizes for analyses differed in dependence of the outcome measure.

**Table 1:** Sample description and group differences.

			AN (n = 34)	BD (n = 17)	Group comparison AN vs. BD		
Demographics					*t/*χ^2^	*p*	*d/r*
Age	In years	*M (SD)*	15.1 (1.6)	15.8 (1.6)	−1.42	.161	0.42
Smoking		*N (%)*	1 (3%)	6 (35%)	10.01	.002**	.44
Weight							
Weight change: admission to pre	In kg	*M (SD)*	7.7 (3.9)	1.0 (2.3)	7.60^a^	<.001**	1.92
BMI pre	In kg/m^2^	*M (SD)*	18.0 (1.1)	24.2 (5.7)	−4.41^a^	<.001**	1.80
BMI post	In kg/m^2^	*M (SD)*	18.4 (1.1)	24.0 (5.7)	−3.90 ^a^	.001**	1.66
Weight change: Pre to post	In kg	*M (SD)*	0.97 (1.1)	-0.44 (1.2)	4.21	<.001**	1.10
Psychometric measures pre							
EDI-2	Drive for thinness—sum	*M (SD)*	30.8 (9.3)	33.6 (4.7)	−1.47^a^	.148	0.36
	Body dissatisfaction—sum	*M (SD)*	38.7 (9.7)	42.9 (7.1)	−1.58	.121	0.47
BIAQ	Total sum	*M (SD)*	36.0 (14.1)	39.3 (8.9)	−1.01	.316	0.26
BDI	Total sum score	*M (SD)*	25.8 (15.3)	39.4 (8.8)	−4.02^a^	<.001**	0.99
Treatment characteristics							
Treatment setting	Inpatient	*N (%)*	25 (74%)	5 (29%)	9.11	.003**	.42
	Day-clinic	*N (%)*	9 (26%)	12 (71%)			
Treatment duration until start of study	in weeks	*M (SD)*	16.4 (11.5)	7.8 (2.8)	4.01^a^	<.001**	0.91
Medication intake	Psychotropic drug intake	*N (%)*	14 (41%)	10 (59%)	1.42	.234	.17
	Contraceptive pill intake	*N (%)*	1 (3%)	2 (12%)	1.59	.207	.18
Comorbidity	Yes (>1 psychiatric diagnoses)	*N (%)*	18 (53%)	11 (65%)	0.64	.424	.11

AN, anorexia nervosa group; BD, body dissatisfied control group; pre, prior to the intervention; post, after intervention; EDI-2, Eating Disorder Inventory-2, short version; BIAQ, Body Image Avoidance Questionnaire; BDI, Beck Depression Inventory. ^a^Corrected for unequal variances. ** p < .01.

### 2.3 Pre–post-intervention measures

Height and weight were recorded at the pre- and post-session of the study and afterwards converted to BMI. The ED psychopathology was assessed with the self-report Eating Disorder Inventory [EDI-2; (47)]. Within the current study, the subscales “body dissatisfaction” and “drive for thinness” were used for analyses, with higher scores implicating higher psychopathology. Furthermore, body avoidance behavior was assessed with the Body Image Avoidance Questionnaire (BIAQ) (48). In order to assess depressive symptoms, the self-report Beck Depression Inventory [BDI-II; (49, 50)] was used, with higher total scores corresponding to more depressive symptoms and scores ranging between 20 and 28 being interpreted as moderate and above 29 as severe depressive symptoms.

### 2.4 Measures during each BE session

For the assessment of subjective stress, participants were asked to rate their level of “stress” using an 11-point Likert scale (0 corresponded to “no stress at all”; 10 to the “maximum imaginable stress”). For the objective stress response, cortisol and sAA levels were assessed in saliva samples. During each BE session, each stress parameter (subjective ratings, cortisol, sAA) was measured four times. Saliva samples were collected using cortisol Salivettes (Sarstedt, Nümbrecht, Germany) to determine free salivary cortisol and sAA as HPA axis and SNS markers, respectively (51). The participants were not allowed to eat, smoke, or drink (except water) for at least 1 h prior to and during the experiment. At each time point, the participants were instructed to keep the swab in the mouth for at least 1 min and to move it inside the oral cavity without biting them. Saliva samples were immediately cooled and stored at −20°C. For analysis, Salivettes were brought to room temperature and centrifuged at 2,000 × g and 20°C for 10 min. At each session, participants were asked for subjective stressors, acute infections, or other relevant day-specific influences to avoid confounding factors on especially stress measures; for analyses, protocols were checked resulting in exclusion of individual samples or even whole sessions, if required.

Salivary cortisol levels were determined using a commercially available cortisol enzyme-linked immunosorbent assay (ELISA) (RE56211, IBL International, Hamburg, Germany) according to the manufacturer’s instructions. All samples obtained from one participant were measured on the same plate. Each sample was assayed in duplicate using a microplate reader (Benchmark Plus™ microplate spectrophotometer, Bio-Rad Laboratories GmbH, Hercules, CA, USA) and quantified against a standard curve generated via four-parameter logistic curve fit. The intra- and inter-assay coefficients of variation (CV) were <10%. Mean values of each duplicate measurement were calculated. Samples with high relative variability (CV >20%) within double measurements were excluded from analyses. Participants were screened for relevant medication intake, such as glucocorticoids and ketoconazole; no participants had to be excluded due to medication. Outliers, defined as raw values deviating more than three standard deviations (SD) from the group mean, were removed, and plausibility checks were carried out. Raw values were log10-transformed prior to statistical analyses to achieve normal distribution.

sAA levels were measured by an in-house enzyme kinetic assay using reagents from DiaSys Diagnostic Systems GmbH. For this evaluation, we followed the description of Nater and Rohleder (51). Briefly, saliva was diluted 1:625 with ultrapure water. Subsequently, the diluted saliva was incubated with a substrate reagent (a-Amylase CC FS; DiaSys Diagnostic Systems) at 37°C (52). A first-absorbance measurement was performed at 405 nm using a Tecan Infinite 200 PRO reader (52). A second measurement was taken after incubation at 37°C for 5 min. The increase in absorbance was converted to sAA concentrations (U/ml) using a standard curve prepared with a “Calibrator f.a.s.” solution (Roche Diagnostics) (52). Participants who smoked or were taking relevant medication, especially adrenergic medication such as catecholamines or ß-blockers (medication: *n* = 0; smokers: *n* = 7), as well as outliers (>3 SDs from group mean) were excluded from the analyses. Afterwards, raw values were log10-transformed to improve normal distribution of the data.

For all three stress measures (rating, cortisol, sAA), besides analyzing the profiles of complete data sets covering 16 samples (T1–T4, each with t1–t4), we calculated stress parameters from raw data in order to analyze specific processes during exposure. Due to different stress reactivity profiles of cortisol and sAA [see, e.g., (53)], the data points used for parameter calculations were adjusted depending on the outcome measure to obtain comparable measures; due to the slower post-stressor release of cortisol (20 min–30 min) compared with sAA, the last cortisol sample (t4) reflects more the stress level at the end of an exposure session than the recovery 30 min afterwards. **Table 2** provides an overview of the analyzed stress parameters.

**Table 2:** Subjective and objective stress parameters.

	Subjective: stress rating	Objective: cortisol	Objective: sAA
**Profile:** course of stress within an exposure session	All 16 samples	All 16 samples	All 16 samples
*Valid samples in analyses: n*	AN = 26, BD = 9	AN = 22, BD = 7	AN = 18, BD = 3
**Anticipation:** pre exposure stress	t1	t1	t1
*Valid samples in analyses: n*	AN = 32, BD = 16	AN = 28, BD = 12	AN = 25, BD = 5
**Within habituation** ^a^: change from start to end of exposure	Difference t3-t1	Difference t4-t1	Difference t3-t1
*Valid samples in analyses: n*	AN = 32, BD = 14	AN = 24, BD = 8	AN = 23, BD = 4
**Recovery** ^a^: stress 30′ post exposure	t4	*Not applicable*	t4
*Valid samples in analyses: n*	AN = 26, BD = 9		AN = 27, BD = 5
**Total release:** release within an exposure session	*Not applicable*	AuCg^b^ t1 to t4	AuCg^b^ t1 to t4
*Valid samples in analyses: n*		AN = 23, BD = 9	AN = 21, BD = 4

AN, anorexia nervosa group; BD, body dissatisfied control group; sAA, salivary alpha amylase; AUC, area under curve. ^a^Due to different reaction times of cortisol and sAA, used data points for parameter calculation were adapted dependent of outcome measure in order to display comparable measures. ^b^AuCg was calculated based on the formula in Pruessner, Kirschbaum (76).

The analyzed stress parameters are marked in bold.

### 2.5 Statistical analyses

Descriptive group differences (AN vs. BD) were tested by *t*-tests or chi-squared tests. For *t*-tests, Cohen’s *d* was used as effect size measure (54). Relevant confounding factors were taken into account in the analyses, if two prerequisites were met: 1. groups differed significantly in the variable tested by *t*-tests (see above), and 2. the variable was significantly associated with the outcome measure (stress rating, cortisol, sAA) using Pearson correlation (*r*). Age, BMI, depressive symptoms, smoking, treatment setting, comorbidity, and medication intake were tested as possible confounding factors; the requirements were met for none of the tested variables, so no covariates were included. To analyze the between and within courses of stress for AN and BD, three-factorial mixed analyses of variance (ANOVA) (1. between-factor “group”: two-staged, AN vs. BD; 2. within-factor “session”: T1–T4; 3. within-factor “time within session”: t1–t4) were run for all stress measures (rating, cortisol, sAA) separately. In order to test differences in the extracted stress parameters (see **Table 2**), two-factorial mixed ANOVAs were calculated with the between-factor “group” (two-staged, AN vs. BD) and the within-factor “session” (four-staged, T1–T4); due to many sAA missing values in the BD group, for these analyses, a dependent one-factorial ANOVA (within-factor “time within session”: t1–t4) was run only for the AN group. Significant effects of ANOVAs were further tested with *post-hoc t-*tests. For ANOVA results, effect sizes were computed as partial *η* ^2^ (*η* ^2^_p_) (54). To analyze the association between subjective and objective stress, correlation analyses were calculated for each time point across both groups. For testing associations between stress (for ratings, cortisol and sAA: single values and stress parameters) and psychopathology (for EDI-2 subscales and BIAQ: pre and post scores as well as difference pre to post), again correlation analyses were run across both groups. Due to missing data, sample size differed between analyses. All analyses were carried out with SPSS (Version 28, SPSS, Chicago, USA). The level of significance was defined as *p* <.05; a correction for multiple testing with Bonferroni was applied if necessary.

## 3 Results

### 3.1 Sample group comparison

As expected from group allocation based on psychiatric diagnoses/symptoms, participants of the AN group had a significantly lower BMI prior to and after the intervention as well as a higher weight gain from admission to the start of the study participation compared with BD. Furthermore, participants of the BD group reported higher scores of depressive symptoms compared with AN, with all of the BD participants having moderate (*n* = 1) or severe (*n* = 16) depressive symptoms according to the BDI sum score in contrast to the AN group with 29% (*n* = 10) or 38% (*n* = 13) having moderate or severe depressive symptoms, respectively. Both groups differed in the percentage being treated in an inpatient or day-clinic setting: while more participants were treated in an inpatient setting in the AN group, in the BD group, more were treated within a day-clinic setting. Furthermore, more adolescents smoked in BD compared with the AN group; all group differences are shown in **Table 1**. The AN and BD groups did not differ in rates of medication intake, with 41% or 59%, respectively, taking mostly one psychotropic drug and *n* = 4 a double medication; medication came from the classes of SSRIs (AN: *n* = 5, BD: *n* = 9) and neuroleptics (AN: *n* = 11, BD: *n* = 3). Furthermore, no group differences were found for age, ED psychopathology in terms of drive for thinness, BD or body image avoidance, and contraceptive pill intake. BD participants of both study centers did also not differ in age, BMI, ED psychopathology, or depressive symptoms (all *p* >.05). Descriptive statistics and complete analyses results are shown in **Table 1**.

### 3.2 Subjective stress ratings

**Figure 1:**
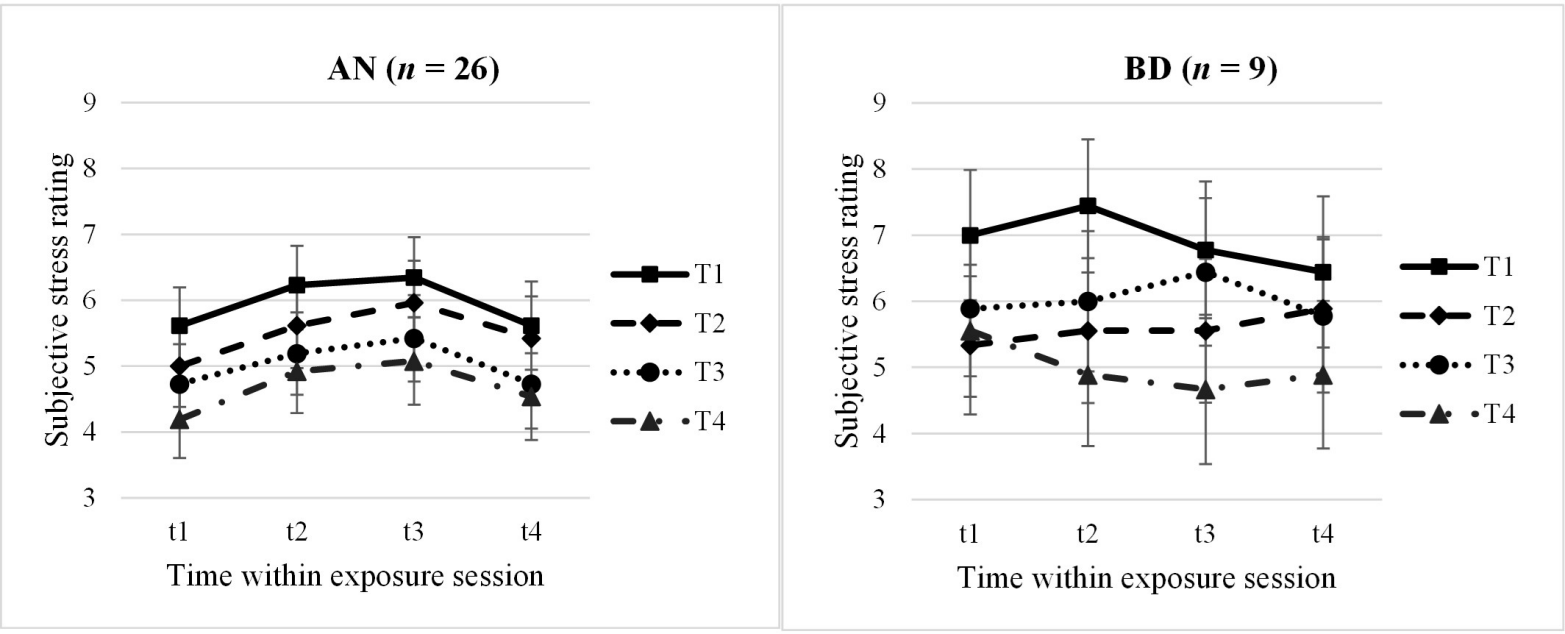
Subjective stress ratings within and throughout the four exposure sessions, split by groups. AN, anorexia nervosa group; BD, body dissatisfied control group. Time within exposure session: t1 = 0′ = prior to start/anticipation, t2 = +10′, t3 = +30′ = end of exposure, t4 = +60′ = 30′ after end/”recovery”. T1–T4 = four exposure sessions within 2.5 weeks. Data are presented as mean +/− SEM.

*N* = 26 AN and *n* = 9 BD adolescents had complete datasets with 16 stress ratings throughout the four exposure sessions, whose profiles were compared within a 2 × 4 × 4 mixed ANOVA. We identified a significant main effect for “Session” with a large effect size (*F* = 9.79, *p* <.001, *n* ^2^_p_ = .49), being interpreted as a between-session habituation: Participants rated their stress levels decreasing from the first to the last session (*M*_T1_ = 6.43, *M*_T2_ = 5.54, *M*_T3_ = 5.52, *M*_T4_ = 4.84), with *post-hoc* tests showing significant higher ratings in the first session and significant lower ratings in the last session compared with the others, respectively (*p* = <.001–.015). No other main or interaction effects reached significance (*p* >.05); specifically, no within-session effect was found. Subjective stress profiles are shown in **Figure 1**, separately for the AN and BD groups.

In a second step, we looked at the subjective stress parameters anticipation (t1), habituation (difference t3 − t1), and recovery (t4) more closely in slightly larger samples due to less missing values using 2 × 4 mixed ANOVAs, respectively. For the subjective anticipatory stress (AN: *n* = 32; BD: *n* = 16; *F* = 2.92, *p* = .045, *n* ^2^_p_ = .17) and the subjective recovery (AN: *n* = 26; BD: *n* = 9; *F* = 3.26, *p* = .035, *n* ^2^_p_ = .24), we again found significant between-session effects, which however would not withstand multiple testing. Participants rated their anticipatory stress significantly lower at the last session compared with the first and third sessions (*p* = .009–.022). Regarding the subjective recovery 30 min after the exposure, a descriptively continuous decrease of stress levels throughout the sessions was found (t4: *M*_T1_ = 6.03, *M*_T2_ = 5.66, *M*_T3_ = 5.25, *M*_T4_ = 4.71). In *post-hoc* analyses, again, t4 at the last exposure session T4 was rated significantly less stressful compared with T1–T3 (*p* = .010–.048).

Neither the ANOVA for the subjective within habituation nor other main or interaction effects reached significance (*p* >.05). Descriptive statistics of subjective stress ratings are listed in the **Supplementary Table S1**; complete results of all ANOVAs regarding subjective stress ratings can be found in the **Supplementary Table S2**.

### 3.3 Cortisol

**Figure 2:**
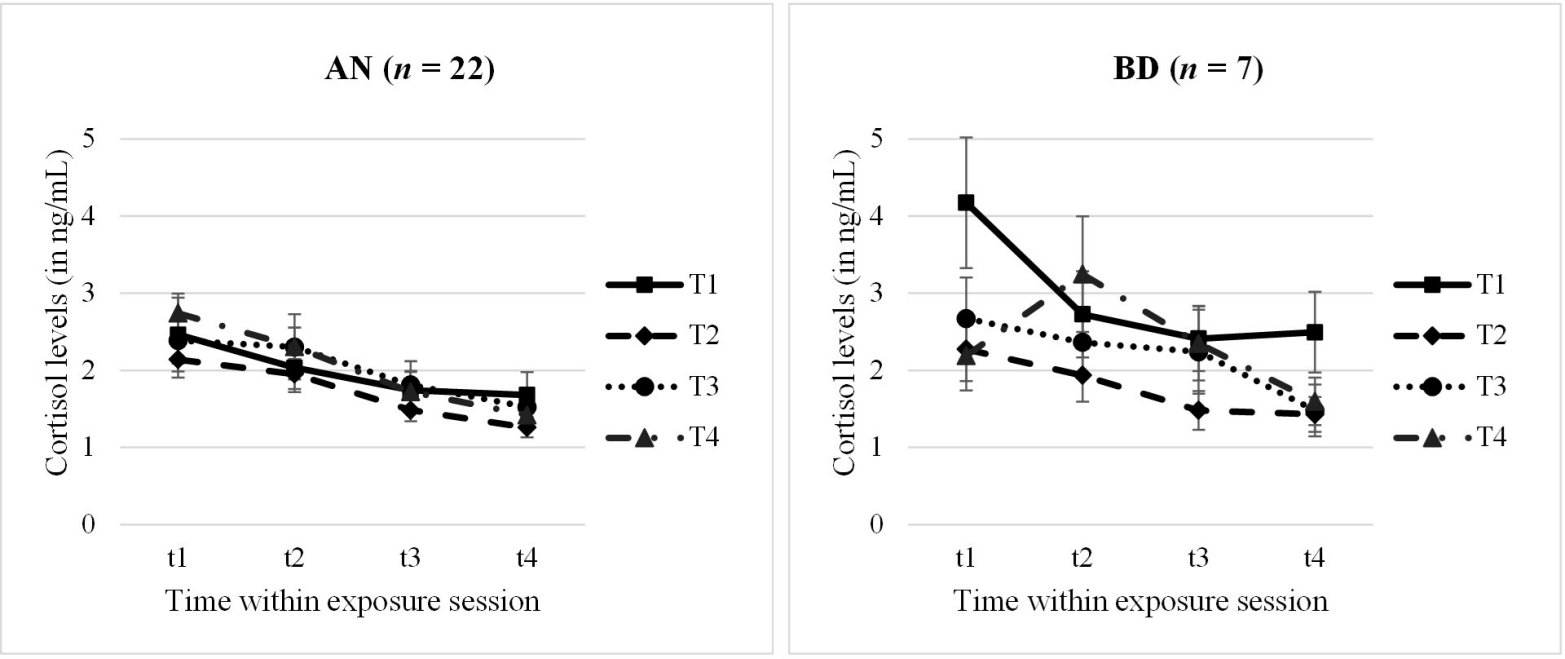
Cortisol levels within and throughout the four exposure sessions, split by groups. AN, anorexia nervosa group; BD, body dissatisfied control group. Time within exposure session: t1 = 0′ = prior to start/anticipation, t2 = +10′, t3 = +30′ = end of exposure, t4 = +60′ = 30′ after end/”recovery”. T1–T4 = four exposure sessions within 2.5 weeks. Data are presented as mean +/− SEM.

Analysing the cortisol profiles (complete data sets for *n* = 22 AN and *n* = 7 BD adolescents) throughout the four exposure sessions within a 2 × 4 × 4 mixed ANOVA, we found a significant main effect for “time within session” with a large effect size (*F* = 13.30, *p* <.001, *n* ^2^_p_ = .62). Demonstrating a within-session habituation effect, cortisol levels decreased significantly from t1 to t4 within each session (*M*_t1_ = 2.53 ng/mL, *M*_t2_ = 2.25 ng/mL, *M*_t3_ = 1.79 ng/mL, *M*_t4_ = 1.54 ng/mL; *p* = <.001–.009). No other main or interaction effect reached significance (*p* >.05); specifically, no between-session effect emerged. Cortisol profiles are shown in **Figure 2**, separately for the AN and BD groups.

Looking at the cortisol parameter anticipation, habituation, and total release (AN: *n* = 23–28; BD: *n* = 8–12) in a second step, 2×4 mixed ANOVAs were run. Here, we found no further significances but only a statistical trend for a between-session effect in cortisol anticipation (*F* = 2.81, *p* = .054, *n* ^2^_p_ = .19) and for a group effect in total release (*F* = 2.75, *p* = .061, *n* ^2^_p_ = .23). At the first session T1, the anticipatory cortisol level was higher than at the other sessions, especially the second and third ones, which seemed descriptively to be driven by the BD group. Furthermore, the BD group tended to have a higher total cortisol release compared with AN. Descriptive statistics of cortisol levels and results of all ANOVAs are presented in **Supplementary Tables S3**, **S4**.

### 3.4 Alpha-amylase

Due to the small sample size in the BD group (*n* = 3–5 dependent on parameter, see **Table 2**) after preprocessing of raw data, quality control, and removing smoking adolescents, sAA analyses were run only within the AN group. An exploratory and preliminary group comparison (AN vs. BD) is reported in **Supplementary Table S5**.

**Figure 3:**
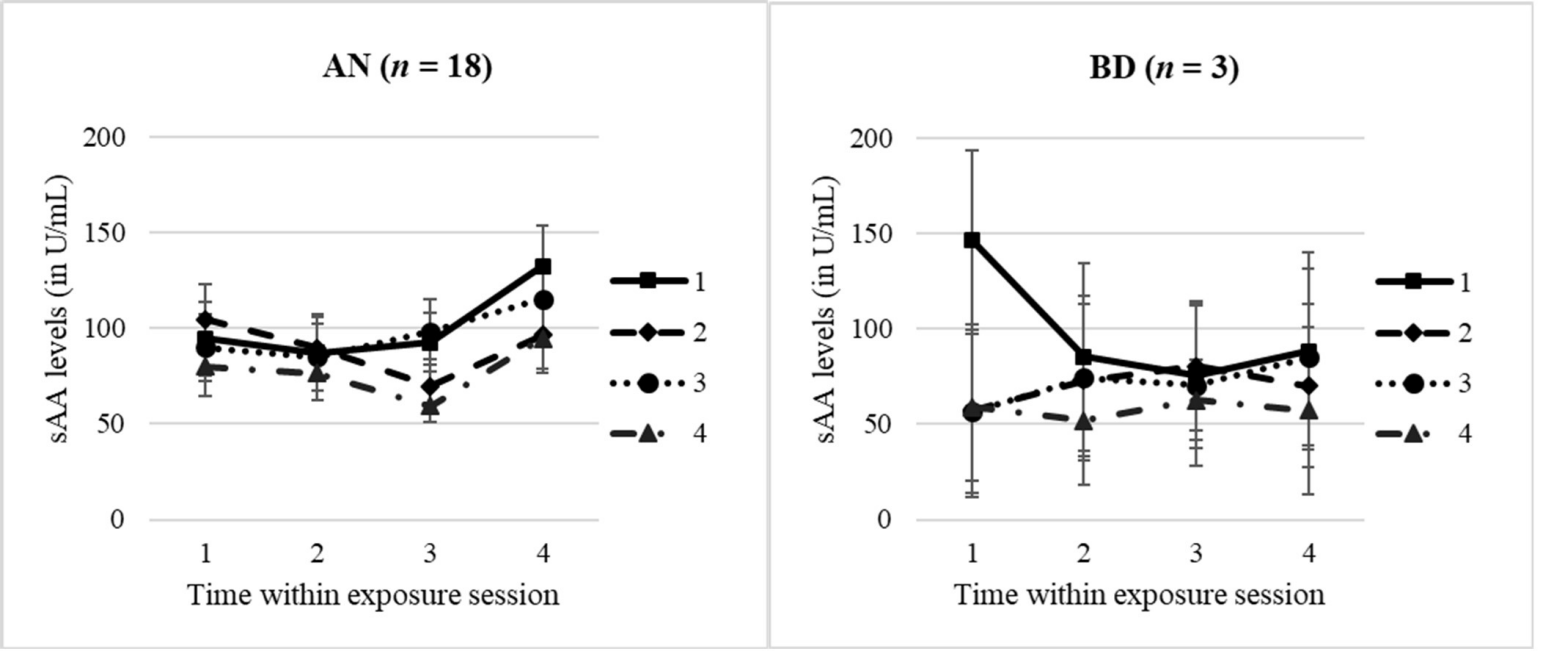
sAA levels within and throughout the four exposure sessions, split by groups. AN, anorexia nervosa group; BD, body dissatisfied control group. Time within exposure session: t1 = 0′ = prior to start/anticipation, t2 = +10′, t3 = +30′ = end of exposure, t4 = +60′ = 30′ after end/”recovery”. T1–T4 = four exposure sessions within 2.5 weeks. Data are presented as mean +/− SEM.

Analysing the sAA profiles (*n* = 18) with a dependent 4 × 4 ANOVA, we found a significant main effect for “time within session” with a large effect size (*F* = 3.68, *p* = .037, *n* ^2^_p_ = .42). For AN, the sAA levels significantly increased 30′ after the exposure session compared with levels during the exposure (t4 vs. t1/t2/t3: *p* = .003–.039). Interestingly, but highly preliminarily due to small sample size, in an exploratory ANOVA comparing the AN vs. BD group (see S5), we identified a significant group effect, with AN adolescents having higher levels compared with BD adolescents 30′ after the exposure (*F* = 9.39, *p* = .005, *n* ^2^ *_p_* = .24). Profiles are shown in **Figure 3**, separately for the AN and BD groups.

Looking at the sAA total release within the AN group, we furthermore identified a significant between-session effect (*F* = 3.86, *p* = .027, *n* ^2^_p_ = .39), with the lowest sAA release in the last exposure session (*post-hoc* tests: T1 vs. T4: *p* = .006; T3 vs. T4: *p* = .004). No other significant effect for any sAA parameter was found, with descriptives of sAA levels and complete results of ANOVAs being presented in **Supplementary Tables S3** and **S4**.

### 3.5 Association of subjective and objective stress measures

In order to investigate the association between subjective and objective stress measures, we conducted correlation analyses across both groups. No significant correlation withstanding multiple testing was found, neither between the subjective and objective measures, respectively, nor between the objective measures themselves.

Only for the within-session habituation (t1–t3 difference) did we find significant correlations between subjective and sAA habituations in two of four sessions with a medium effect size (T1: *r* = .37, *p* = .020; T3: *r* = .38, *p* = .026): A greater subjective stress reduction was associated with a larger decrease in sAA levels within an exposure session. However, results should be interpreted with caution, because they did not withstand multiple testing and were only found at two sessions, which questions the validity.

### 3.6 Association of psychopathology and stress

Looking at associations of ED psychopathology with subjective stress ratings in correlation analyses across both groups, we identified large and persistent positive correlations (*r* = .40–.73, *p* = <.001–.009) throughout all time points within a session (t1–t4) across all sessions (T1–T4): Adolescents with higher BD and higher drive for thinness (EDI-2 subscale scores) as well as more body image avoidance (BIAQ score) before and after the intervention rated their stress levels consistently higher. Furthermore, only in patients with AN but not the BD group were higher stress ratings significantly associated with higher depressive symptoms (*p* = <.001–.013).

Looking at the associations of psychopathology and cortisol levels across both groups, we found a stronger symptom reduction in BD (difference pre to post intervention) being associated with higher cortisol levels at the end of the intervention: A greater improvement was associated with higher cortisol levels in the last exposure session (t2: *r* = .51, *p* = .001; t3: *r* = .37, *p* = .028) as well as with a higher total cortisol release in T3 and T4 (T3: *r* = .38, *p* = .031; T4: *r* = .48, *p* = .004); a similar pattern was found for the reduction of other symptoms and total release but did not reach significance (“Drive for thinness”: *r* = .33, *p* = .055; Body image avoidance: *r* = .31, *p* = .079). For sAA or any other psychopathologic parameter, we did not find a significant correlation.

## 4 Discussion

Within a controlled design comparing adolescents with AN to adolescents with high BD, we investigated the subjective and objective stress response to a computer-based BE. In partial agreement with our hypotheses, we found a between-session habituation for subjective stress ratings and sAA levels as well as a within-session habituation for cortisol levels, but no concurrent between- and within-session habituation for any measure. Furthermore, a higher psychopathology corresponded to higher stress ratings. In contrast to our hypotheses, there were no group effects regarding subjective or objective stress levels or reactivity. In total and throughout the analyses, both groups were more similar than different. Due to small sample sizes especially in the objective stress measures of the BD group, however, results should be interpreted with care.

Regarding subjective stress ratings, as hypothesized, we found a between-session habituation with decreasing subjective stress throughout all sessions, with highest ratings in the first and lowest in the fourth session for both groups. For the anticipatory stress and the recovery 30′ after the end of an exposure session, between-session habituation was confirmed, with effects especially being driven by lower ratings in the last exposure session. Results are interpreted in terms of adolescents getting more used to their body image and feeling less stressed by confrontation with a picture of one’s own body. This is in line with former studies showing an affective between-session habituation by BE interventions (22, 27).

We did not find a within-session habituation effect for subjective stress in terms of an expected continuous decrease, but instead a non-significant increase from the start to the end of a session followed by a stress decrease 30′ later. This pattern is comparable with findings from other guided BE interventions and therefore might be explained by theories proposing different working mechanisms for pure compared with guided BE (21, 22). Within the guided BE used here, t2 corresponded to the focus of the torso (abdomen, hips, upper thigh), which is known as a “critical” body part activating BD, and might explain the higher stress ratings compared with t1. This is in contrast to pure BE, which initially focuses on the worst body part(s) due to an attentional bias (55, 56) and then might continue with a decreasing focus on stress/anxiety provoking parts, which is accompanied by decreasing stress levels.

Between-session habituation found here has been shown to be relevant for treatment outcome in a food exposure study and hypothesized as more relevant for treatment effects compared with within-session habituation, so current results are promising (30). Furthermore, a reduction in subjective stress after just a few sessions appears to be clinically relevant, as this could make it easier for patients to repeatedly use this highly relevant and effective, but stressful intervention. A regular active confrontation with the own body, recommended initially under therapeutic guidance followed by a more and more independent practice of BE (14, 18), allows a modification of the body image disturbance as a relevant maintaining factor of AN (1) as well as a risk factor for the development of an ED in body dissatisfied adolescents (11, 12).

We found strong and continuous positive associations of ED psychopathology with subjective stress ratings, so adolescents with more ED symptoms feel more stressed during a confrontation with one’s one body. Interestingly, only in patients with AN did we identify an association of depressive symptoms and stress ratings, which might be explained by the larger variance within this group: the BD group consisted of mainly highly depressed adolescents, which is in line with former studies showing associations of BD and depressive symptoms (8–10); in contrast, within the AN group, depressive symptoms differed clearly. It is hypothesized that adolescents with both AN and depressive symptoms might feel especially stressed by this intervention, which should be considered in the clinical implementation modalities of BE (e.g., more guided sessions at the beginning, gradual approach).

For cortisol, we found a within-session habituation showing continuously decreasing cortisol levels from the first to the last sample within each exposure session. This stands in contrast to a former study showing no within habituation during BE in patients with AN (32) but corresponds to a BE study with patients with BN and high BD (22). So, adolescents seem to get used to the exposure and habituate physiologically during a session, which is a promising result especially against the background of the subjective stress increase described earlier. The discrepancy to the subjective stress course might be explained by the cognitive–affective activation of BD, which could be simplified or “misinterpreted” as stress by the participants that is not equivalent to physiological stress.

For cortisol, we did not find a between-session habituation effect. Similarly, Díaz-Ferrer et al. also found an indication of a between-session habituation only in the pure and not in the guided exposure condition (22). This could be explained here by the low number of exposure sessions and the short time span of 2.5 weeks, so biological stress systems might require a longer time or several exposure sessions until habituation could be observed. This is supported by Schmalbach et al., who showed that despite “recovery” of basal HPA functionality, irregularities in the HPA reactivity still remain after weight gain indicating a longer-term “recovery” process here (36).

Interestingly, investigating the association of psychopathology and cortisol, we found higher cortisol levels in the last exposure session(s) corresponding to a greater ED symptom reduction from pre to post intervention. This finding is counterintuitive and might be interpreted as an effect of social desirability or dissimulation of patients with especially still high objective stress levels at the end of the intervention, rating a greater symptom change after the completed intervention. However, more research and an explicit assessment of social desirability is needed to validate this hypothesis.

Due to the very small sample size in the BD group, only the sAA levels of the AN group could be analyzed and interpreted. Hinting to a between-session habituation effect and fitting our hypothesis, we found a lower total sAA release in the last exposure session compared with former sessions. This might reflect familiarization of the adolescents with the BE across the intervention and corresponds to our subjective habituation results. So, across the four BE sessions, subjective stress and total sAA release both decrease; however, habituation seems to take place at different speeds, with biological reaction delaying in comparison with the subjective one.

Also comparable with the subjective stress results, we did not identify a within-session effect for sAA in AN patients only. This might be explained by the pre-described blunted SNS activity in patients with AN reflected by an attenuated sAA response to a stressor (37, 42).

Interestingly, while we did not find any significant change in the sAA levels during BE, we identified an sAA increase 30′ afterwards. Despite of the small sample of BD adolescents with valid sAA levels, a significant difference at t4 was found between patients with AN and adolescents of the BD group in a preliminary group comparison, so it could be speculated that this pattern might be an AN-specific “post-processing” effect. Rumination about body weight, figure, and food is a common symptom and discussed as a maintaining factor of AN (57, 58). While rumination about food was shown to be associated with aspects of malnutrition, rumination about body weight and shape was more associated with affect (59), especially with negative affect (57, 60). Furthermore, rumination was hypothesized as a (maladaptive) cognitive strategy for emotion regulation of patients with AN (58, 61). In the current study, we might have further support for this idea: higher stress after BE shown by sAA levels might be regulated by rumination, which might in turn explain the discrepancy of sAA and subjective stress ratings at this time point.

Group comparisons between AN and BD showed mainly no differences for both subjective and objective stress. On the one hand, this could be based on a statistical effect due to the unequal sample size and the small BD group; so, the current results and our hypotheses and conclusions should therefore be interpreted with care and possible confounding factors such as depressive symptoms and medications effects (see limitations) have to be kept in mind. On the other hand, however, this finding could also hint to a high similarity of both groups, which is supported by similar self-rated ED symptoms including BD, drive for thinness, and body image avoidance. Therefore, one might speculate that BE is equally stressful for both groups, which is in turn supported by the high and consistent positive correlations between psychopathology and subjective stress ratings found across BE and the groups. So, high BD might make the groups more similar than assumed when simply looking at the psychiatric diagnoses used for group allocation and hint to a transdiagnostic phenomenon of BD. Compared with other stress research with healthy subjects, the current reactivity data are in a comparable range and not higher or lower than in healthy subjects [e.g., (62)]. In order to enable an even better classification of the current objective stress levels, baseline data would be desirable in future studies, as the initial measurement (t1) of the present study must be regarded as an anticipation condition.

Regarding the objective stress measure cortisol, the non-existent group difference might further be explained by the main symptomatology of the BD group in terms of depression as all BD participants had a diagnosis of depression and moderate to severe depressive symptoms in the BDI-II: for AN and depression, similar adaptations of the HPA axis (and the SNS) are described in terms of higher cortisol levels [e.g., for AN: (38, 63), for depression: (64, 65)] and a blunted stress reactivity [e.g., for AN: (37), for depression: (66–68)]. Chronic and repetitive stress is a major risk factor for the development of depressive symptoms (68, 69). It is known that chronic stress causes an excessive release of cortisol as an adaptive reaction to stressful situations; however, if chronic stress persists or cortisol levels persist to be high, this adaptivity is exhausted and reactivity to acute stress is blunted, which we found in our data (67, 68). Nevertheless, descriptively (and in parts as a statistical trend), we found higher levels at the very first cortisol sample (t1 in T1) and a tendency towards higher total cortisol release in the BD group. The high cortisol release at the first session in the BD group might be explained by the fact that these adolescents were hardly familiar with body image-related interventions due to other therapeutic foci of their treatment, whereas most adolescents with AN were more familiar with body themes and interventions. The higher total release might represent an even stronger blunted HPA reactivity in AN compared with depression. Furthermore, looking at the courses of the subjective and in less extent objective stress measures for the AN and BD groups, one might speculate different habituation processes: while the AN group showed a similar course for each session, the BD group courses were different for each session, which should be interpreted with caution against the background of the small BD sample. More studies with larger sample sizes are needed to validate these first impressions.

Except for a slight hint towards a subjective and sAA relation regarding within-session habituation, we did not identify associations between subjective and objective stress measures. This discrepancy has also been reported in former BE studies (25, 27, 32) or other stress research [e.g., (70, 71)]. This finding might underline that the assessment of subjective and objective stress measures represents different modalities, at different levels of the CNS or dependent systems, which for example might show a different temporal trajectory with one system changing earlier or later than the other. Another explanation for the inconsistency of subjective and objective stress might be that BE has been shown to activate different emotions (72–74), which in turn might activate the physiological stress systems differently. Furthermore, one might speculate that the rated “stress” might represent a mixture of different cognitive and affective processes. In order to obtain proof, further studies with a more differentiated assessment of emotions and especially cognitions are important.

Regarding the non-association of cortisol and sAA, further research is urgently necessary, due to the small number of studies to date that examine cortisol and sAA together in EDs: In patients with AN, Monteleone et al. reported an asymmetry of both systems and we also did not find any associations (42). For externalizing and internalizing behavior problems, a dysregulation of the HPA axis and the SNS has been found in adolescents; however, there are mixed results regarding the way of dysregulation (43). This might fit with the postulations of Bauer et al., stating that a dysregulation in both systems (additive hypothesis) but also a dysregulation in only one of both systems (interactive hypothesis) may lead to behavior problems, which has to be investigated in future disorder-specific research (75).

For clinical practice, on the one hand, the present results are relevant for the treatment of adolescents with AN: first, the present results can be used in psychoeducation for BE and interpreted as objective and subjective habituation, which may make BE appear more manageable for patients. Furthermore, the current study presents the use of photos of one’s own body as a further variant of BE that shows comparable stress patterns to former studies of BE *in vivo* or virtual reality; BE using photos could be an easily applicable method for clinical practice, also in self-management and in the home environment, for which further studies are needed. On the other hand, results are relevant for all other patients with “comorbid” BD: Based on the current and previous findings, clinicians should consider BD in the diagnostic and treatment processes, especially in patients with depression. BE could also be used in these patients as a method to reduce BD and possibly thereby improve self-esteem and self-efficacy, which should be investigated in future studies.

### 4.1 Limitations

First of all and contrary to our study protocol, sample sizes differed between groups and parameters, resulting in partly small and unequal sample sizes: A smaller sample size in the BD group was related to a lower inclusion and consent rate to study participation in this group which might be explained by disorder-associated phenomena (e.g., loss of energy and interest, low self-esteem), an observed high comorbidity between depression and abnormal eating behaviors resulting in non-inclusion to preserve distinct groups, and the “unfamiliar” treatment module of body exposure for patients with emotional burden and no diagnosed ED. Furthermore, due to the intense study design with six sessions within 2.5 weeks and 16 saliva samples, including four samples after the exposure sessions when participants have returned to clinic routine, single saliva samples were missing, resulting in a reduced sample size for objective stress analyses; the higher smoking rating in the BD group further reduced sAA sample sizes for these analyses. Secondly, the evaluated BE model including four sessions of exposure within a period of approximately 2 weeks here might be too short for adaptations in neurobiological systems to occur, so effects should be followed over a longer period of time and maybe more sessions in future studies. Furthermore, BD was assessed at the beginning of the study but not at admission; investigating the time course of BD could provide further insight into its development and its impact on treatment modules, including BE, in future studies. Thirdly, we included a wide patients’ age range from young adolescents to nearly adults: in former studies, objective markers have been shown to be associated with age (which did not differ between groups or was associated with outcome measures here and therefore not controlled for) and pubertal status (which was not assessed here); in future studies with larger sample size, it would be interesting to compare different age and puberty groups to get more insights in developmental processes. Besides age, different main and comorbid diagnoses within the small BD group led to a high heterogeneity within this group, which again underlines the need for a larger group and the possibility of more group-homogenous analyses, e.g., regarding depressive symptoms, which might affect cortisol response. Psychopharmacological medication could have had effects on visual and emotional perception and therefore stress ratings. Similarly, medication could affect cortisol and sAA basal levels, however, as individual profiles rather than basal levels were examined, those effects should have been less interfering. The lower BMI of the AN group could similarly have an impact on the tested behavioral and biological markers—an interfering factor which cannot be ruled out in studies with anorexia patients. As patients with AN had reached a weight above the 10th age percentile in order to be included in the study, results are not generalizable to more underweight participants. Finally, no healthy control group was surveyed, so no assessment and comparison of BD and preoccupation with weight and physical appearance in the general population were possible; the current results might be further influenced by the psychiatric diagnoses that were present in addition to the BD within the clinical BD group. In future studies, a control group of adolescents without psychopathology should be included to assess these aspects and to evaluate the BE program in this group of participants.

## 5 Conclusion

Summarizing the current results, we identified subjective and objective habituation processes within a four-session computer-based BE intervention in adolescents with AN and high BD. Both groups get more and more familiar and less stressed by confrontation with images of their own body, which in turn might facilitate to continue the effective intervention of BE and therefore reduce body image disturbance as a relevant risk and maintaining factor for EDs. BD is hypothesized as being a transdiagnostic phenomenon, and related interventions should be incorporated in treatment programs independent of an ED diagnosis. Future studies expanded by follow-up measurements are needed to validate the current findings in the short and long terms and to further investigate the role of cognitions and other emotions to understand the findings presented here.

## Data Availability

The raw data supporting the conclusions of this article will be made available by the authors, without undue reservation.
