# Supplementary material for: Subjective and objective stress during body exposure: a comparison of adolescents with anorexia nervosa versus high body dissatisfaction
Source: Front Psychiatry. 2025 Jan 20;15:1452923. doi: 10.3389/fpsyt.2024.1452923 (PMC11788343; doi:10.3389/fpsyt.2024.1452923)
Supplement: Supplementary file 1 [file DataSheet1.pdf]

*Supplementary Material to*

# Subjective and objective stress during body exposure: a comparison of adolescents with anorexia nervosa versus high body dissatisfaction

**Table S1. Descriptive statistics of subjective stress ratings.** ........ 2

**Table S2. ANOVA results for subjective stress.** ........ 3

**Table S3. Descriptive cortisol levels in ng/ml.** ........ 4

**Table S4. ANOVA results for cortisol levels.** ........ 5

**Table S5. Descriptive sAA levels in U/ml.** ........ 6

**Table S4. ANOVA results for sAA levels within AN group.** ........ 7

**Table S5. Exploratory and preliminary ANOVA results for sAA levels with AN vs. BD group comparison.** ........ 8

**Table S6. Correlation analyses for stress parameters.** ........ 9

**Table S1. Descriptive statistics of subjective stress ratings.**

| **Subjective stress rating** | | AN | | BD | | Group comparison AN vs. BD | | |
|---|---|---|---|---|---|---|---|---|
| Exposure session | Time within session | *n* | *M(SD)* | *n* | *M(SD)* | *t* | *p* | *d* |
| T1 | t1 | 34 | 5.6 (3.1) | 17 | 6.8 (1.9) | -1.72 [a] | .092 | 0.44 |
| | t2 | 34 | 6.2 (3.2) | 17 | 7.4 (1.8) | -1.61 [a] | .113 | 0.40 |
| | t3 | 34 | 6.5 (3.2) | 16 | 7.1 (2.3) | -0.75 [a] | .460 | 0.20 |
| | t4 | 28 | 5.8 (3.5) | 14 | 6.0 (2.7) | -0.20 | .842 | 0.07 |
| T2 | t1 | 34 | 5.3 (3.2) | 17 | 5.8 (2.4) | -0.70 | .545 | 0.18 |
| | t2 | 34 | 6.0 (3.2) | 17 | 6.2 (2.8) | 0.16 | .826 | 0.05 |
| | t3 | 34 | 6.2 (3.1) | 17 | 6.5 (2.7) | -.039 | .695 | 0.12 |
| | t4 | 29 | 5.7 (3.3) | 16 | 5.9 (2.6) | 0.22 | .826 | 0.07 |
| T3 | t1 | 34 | 5.2 (3.2) | 17 | 6.2 (2.5) | -1.21 | .234 | 0.36 |
| | t2 | 34 | 5.6 (3.2) | 17 | 6.5 (2.8) | -0.90 | .374 | 0.27 |
| | t3 | 34 | 5.8 (3.3) | 17 | 6.9 (2.7) | -1.24 | .222 | 0.37 |
| | t4 | 28 | 5.0 (3.6) | 15 | 5.7 (2.7) | -0.80 [a] | .430 | 0.23 |
| T4 | t1 | 32 | 4.8 (3.3) | 16 | 5.9 (1.9) | 1.51 [a] | .139 | 0.40 |
| | t2 | 32 | 5.5 (3.4) | 16 | 5.9 (2.4) | -0.40 [a] | .689 | 0.11 |
| | t3 | 32 | 5.7 (3.4) | 15 | 5.6 (3.0) | 0.06 | .956 | 0.02 |
| | t4 | 30 | 4.9 (3.3) | 13 | 5.6 (3.1) | -0.69 | .955 | 0.23 |

*Notes*. Time within exposure session: t1 = 0’ = prior to start / anticipation, t2 = +10’, t3 = +30’ = end of exposure, t4 = +60’ = 30’ after end / “recovery”. T1-T4 = four exposure sessions within 2.5 weeks. [a]corrected for unequal variances.

**Table S2. ANOVA results for subjective stress.**

| | ***F*** | ***p*** | $\eta_p^2$ |
|---|---|---|---|
| **Subjective stress profiles: 2x4x4 ANOVA** (AN: *n* = 26; BD: *n* = 9) | | | |
| ME group | 0.27 | .607 | .01 |
| ME session | 9.79 | <.001** | .49 |
| ME time within session | 1.14 | .347 | .10 |
| IA group x session | 1.36 | .274 | .12 |
| IA group x time within session | 1.23 | .317 | .11 |
| IA session x time within session | 1.19 | .341 | .30 |
| IA group x session x time within session | 0.90 | .543 | .24 |
| **Subjective anticipation: 2x4 ANOVA** (AN: *n* = 32; BD: *n* = 16) | | | |
| ME group | 1.28 | .265 | .03 |
| ME session | 2.92 | .045* | .17 |
| IA group x session | 0.63 | .600 | .04 |
| **Subjective within-habituation: 2x4 ANOVA** (AN: *n* = 32; BD: *n* = 14) | | | |
| ME group | 3.65 | $.063^{+}$ | .08 |
| ME session | 1.31 | .283 | .09 |
| IA group x session | 2.35 | $.086^{+}$ | .14 |
| **Subjective recovery: 2x4 ANOVA** (AN: *n* = 26; BD: *n* = 9) | | | |
| ME group | 0.32 | .579 | .01 |
| ME session | 3.26 | $.035^{+}$ | .24 |
| IA group x session | 0.57 | .640 | .05 |

*Notes*. AN: Anorexia nervosa group, BD: body dissatisfied control group. ME = main effect, IA = interaction effect.

**Table S3. Descriptive cortisol levels in ng/ml.**

| **Cortisol** | | AN | | BD | | Group comparison AN vs. BD | | |
|---|---|---|---|---|---|---|---|---|
| Exposure session | Time within session | *n* | *M(SD)* | *n* | *M(SD)* | *t* | *p* | *d* |
| T1 | t1 | 33 | 2.90 (2.33) | 17 | 3.54 (2.93) | -0.85 | .399 | 0.25 |
| | t2 | 32 | 2.18 (1.24) | 17 | 2.97 (1.24) | -1.26 [a] | .223 | 0.46 |
| | t3 | 33 | 2.11 (1.40) | 16 | 2.39 (2.06) | -0.57 | .569 | 0.18 |
| | t4 | 32 | 1.95 (1.53) | 15 | 2.31 (2.29) | -0.64 | .525 | 0.20 |
| T2 | t1 | 33 | 2.58 (1.67) | 16 | 1.87 (1.34) | 1.49 | .142 | 0.45 |
| | t2 | 33 | 2.38 (1.18) | 16 | 1.77 (1.13) | 1.72 | .092 | 0.52 |
| | t3 | 33 | 1.82 (1.11) | 16 | 1.61 (1.08) | 0.61 | .542 | 0.19 |
| | t4 | 30 | 1.30 (0.61) | 15 | 1.23 (0.72) | 0.38 | .707 | 0.12 |
| T3 | t1 | 31 | 2.20 (1.27) | 15 | 2.08 (1.50) | 0.28 | .785 | 0.09 |
| | t2 | 34 | 2.64 (1.62) | 15 | 2.16 (1.92) | 0.91 | .369 | 0.28 |
| | t3 | 33 | 1.94 (1.10) | 15 | 2.03 (2.29) | -0.15 [a] | .886 | 0.06 |
| | t4 | 32 | 1.72 (1.14) | 13 | 1.28 (0.78) | 1.29 | .204 | 0.42 |
| T4 | t1 | 32 | 2.60 (1.41) | 14 | 2.02 (0.96) | 1.38 | .176 | 0.44 |
| | t2 | 30 | 2.34 (1.28) | 15 | 2.88 (2.88) | -0.69 [a] | .503 | 0.27 |
| | t3 | 29 | 1.85 (1.02) | 14 | 2.39 (2.23) | -0.86 [a] | .405 | 0.35 |
| | t4 | 30 | 1.50 (0.90) | 12 | 1.60 (1.16) | -0.30 | .763 | 0.10 |

*Notes*. Time within exposure session: t1 = 0’ = prior to start / anticipation, t2 = +10’, t3 = +30’ = end of exposure, t4 = +60’ = 30’ after end / “recovery”. T1-T4 = four exposure sessions within 2.5 weeks.
[a]corrected for unequal variances.

**Table S4. ANOVA results for cortisol levels.**

| | $F$ | $p$ | $\eta_p^2$ |
|---|---|---|---|
| **Cortisol profiles: 2x4x4 ANOVA** (AN: *n* = 22; BD: *n* = 7) | | | |
| ME group | 0.22 | .640 | .01 |
| ME session | 2.87 | $.057^{+}$ | .26 |
| ME time within session | 13.30 | <.001** | .62 |
| IA group x session | 0.72 | .548 | .08 |
| IA group x time within session | 0.24 | .868 | .03 |
| IA session x time within session | 0.98 | .486 | .32 |
| IA group x session x time within session | 1.05 | .439 | .33 |
| **Cortisol anticipation: 2x4 ANOVA** (AN: *n* = 28; BD: *n* = 12) | | | |
| ME group | 0.53 | .470 | .01 |
| ME session | 2.81 | $.054^{+}$ | .19 |
| IA group x session | 1.47 | .240 | .11 |
| **Cortisol within-habituation: 2x4 ANOVA** (AN: *n* = 24; BD: *n* = 8) | | | |
| ME group | 0.33 | .805 | .03 |
| ME session | 1.41 | .261 | .13 |
| IA group x session | 0.04 | .843 | <.01 |
| **Cortisol total release: 2x4 ANOVA** (AN: *n* = 23; BD: *n* = 9) | | | |
| ME group | 2.75 | $.061^{+}$ | .23 |
| ME session | 0.87 | .468 | .09 |
| IA group x session | 1.27 | .269 | .04 |

*Notes*. AN: Anorexia nervosa group, BD: body dissatisfied control group. ME = main effect, IA = interaction effect.

**Table S5. Descriptive sAA levels in U/ml.**

| sAA | | AN | | BD | | Group comparison AN vs. BD | | |
|---|---|---|---|---|---|---|---|---|
| Exposure session | Time within session | *n* | *M(SD)* | *n* | *M(SD)* | *t* | *p* | *d* |
| T1 | t1 | 33 | 96.5 (65.7) | 10 | 132.9 (114.4) | -1.00 [a] | .359 | 0.46 |
| | t2 | 33 | 88.3 (77.7) | 10 | 103.3 (81.5) | -0.53 | .600 | 0.19 |
| | t3 | 31 | 92.9 (58.3) | 9 | 80.2 (92.8) | 0.50 | .620 | 0.19 |
| | t4 | 32 | 158.5 (174.1) | 9 | 81.2 (65.6) | 1.30 | .202 | 0.49 |
| T2 | t1 | 32 | 88.4 (67.0) | 9 | 63.7 (59.3) | 1.00 | .322 | 0.38 |
| | t2 | 33 | 85.4 (60.7) | 10 | 71.8 (47.4) | 0.65 | .520 | 0.23 |
| | t3 | 33 | 74.1 (51.3) | 9 | 76.0 (53.3) | -0.10 | .921 | 0.04 |
| | t4 | 31 | 103.9 (69.0) | 10 | 103.8 (75.1) | 0.00 | .999 | 0.00 |
| T3 | t1 | 29 | 91.9 (68.1) | 7 | 69.5 (55.8) | 0.80 | .427 | 0.34 |
| | t2 | 32 | 78.7 (63.0) | 8 | 61.3 (77.0) | 0.67 | .508 | 0.26 |
| | t3 | 32 | 97.3 (72.8) | 7 | 62.9 (51.6) | 1.18 | .246 | 0.49 |
| | t4 | 31 | 103.6 (69.3) | 7 | 63.1 (73.4) | 1.38 | .176 | 0.58 |
| T4 | t1 | 30 | 81.0 (59.8) | 9 | 92.5 (89.0) | -0.45 | .653 | 0.17 |
| | t2 | 29 | 71.8 (52.5) | 9 | 63.9 (61.6) | 0.38 | .707 | 0.14 |
| | t3 | 30 | 68.0 (52.1) | 9 | 55.9 (38.9) | 0.64 | .524 | 0.25 |
| | t4 | 31 | 134.8 (179.9) | 8 | 76.1 (92.3) | 0.89 | .381 | 0.35 |

*Notes*. Time within exposure session: t1 = 0’ = prior to start / anticipation, t2 = +10’, t3 = +30’ = end of exposure, t4 = +60’ = 30’ after end / “recovery”. T1-T4 = four exposure sessions within 2.5 weeks. [a]corrected for unequal variances.

**Table S4. ANOVA results for sAA levels within AN group.**

| | ***F*** | ***p*** | $\eta_p^2$ |
|---|---|---|---|
| **sAA profiles: 4x4 ANOVA** (AN: *n* = 18) | | | |
| ME session | 1.50 | .253 | .23 |
| ME time within session | 3.67 | .037* | .42 |
| IA session x time within session | 2.52 | $.092^{+}$ | .72 |
| **sAA anticipation: 1x4 ANOVA** (AN: *n* = 25) | | | |
| ME session | 1.81 | .175 | .20 |
| **sAA within-habituation: 1x4 ANOVA** (AN: *n* = 23) | | | |
| ME session | 1.95 | .154 | .23 |
| **sAA recovery: 1x4 ANOVA** (AN: *n* = 27) | | | |
| ME session | 1.48 | .246 | .16 |
| **sAA total release: 1x4 ANOVA** (AN: *n* = 21) | | | |
| ME session | 3.86 | .027* | .39 |

*Notes*. AN: Anorexia nervosa group, BD: body dissatisfied control group. ME = main effect, IA = interaction effect.

**Table S5. Exploratory and preliminary ANOVA results for sAA levels with AN vs. BD group comparison.**

| | ***F*** | ***p*** | $\eta_p^2$ |
|---|---|---|---|
| **sAA profiles: 2x4x4 ANOVA** (AN: *n* = 18; BD: *n* = 3) | | | |
| ME group | 0.25 | .625 | .01 |
| ME session | 2.04 | .147 | .26 |
| ME time within session | 1.24 | .327 | .18 |
| IA group x session | 0.34 | .794 | .06 |
| IA group x time within session | 0.51 | .682 | .08 |
| IA session x time within session | 2.15 | .115 | .64 |
| IA group x session x time within session | 2.28 | .100 | .65 |
| **sAA anticipation: 2x4 ANOVA** (AN: *n* = 25; BD: *n* = 5) | | | |
| ME group | 1.49 | .232 | .05 |
| ME session | 4.44 | .012* | .34 |
| IA group x session | 5.35 | .005** | .38 |
| **sAA within-habituation: 2x4 ANOVA** (AN: *n* = 23; BD: *n* = 4) | | | |
| ME group | 0.11 | .748 | .00 |
| ME session | 2.40 | .094$^{+}$ | .24 |
| IA group x session | 3.92 | .021* | .34 |
| **sAA recovery: 2x4 ANOVA** (AN: *n* = 27; BD: *n* = 5) | | | |
| ME group | 9.39 | .005** | .24 |
| ME session | 0.81 | .500 | .08 |
| IA group x session | 0,15 | .928 | .02 |
| **sAA total release: 2x4 ANOVA** (AN: *n* = 21; BD: *n* = 4) | | | |
| ME group | 1.28 | .270 | .05 |
| ME session | 1.91 | .158 | .22 |
| IA group x session | 0.22 | .882 | .03 |

*Notes*. AN: Anorexia nervosa group, BD: body dissatisfied control group. ME = main effect, IA = interaction effect.

**Table S6. Correlation analyses for stress parameters.**

| Anticipation | T1 | T2 | T3 | T4 |
|---|---|---|---|---|
| Subjective stress x cortisol | *r* = .20, *p* = .199 | *r* = .22, *p* = .178 | *r* = -.07, *p* = .658 | *r* = -.11, *p* = .512 |
| Subjective stress x sAA | *r* = .01, *p* = .948 | *r* = -.03, *p* = .871 | *r* = -.09, *p* = .609 | *r* = -.10, *p* = .530 |
| Cortisol x sAA | *r* = .26, *p* = .103 | *r* = -.04, *p* = .828 | *r* = .02, *p* = .914 | *r* = .03, *p* = .857 |
| **Within-habituation** | **T1** | **T2** | **T3** | **T4** |
| Subjective stress x cortisol | *r* = .01, *p* = .958 | *r* = .23, *p* = .170 | *r* = .16, *p* = .359 | *r* = -.06, *p* = .752 |
| Subjective stress x sAA | *r* = .37*, *p* = .020 | *r* = .16, *p* = .322 | *r* = .38*, *p* = .026 | *r* = -.16, *p* = .340 |
| Cortisol x sAA | *r* = -.07, *p* = .671 | *r* = -.19, *p* = .271 | *r* = -.05, *p* = .788 | *r* = -.10, *p* = .582 |
| **Recovery** | **T1** | **T2** | **T3** | **T4** |
| Subjective stress x cortisol | *r* = .03, *p* = .853 | *r* = .25, *p* = .146 | *r* = .12, *p* = .512 | *r* = -.08, *p* = .638 |
| Subjective stress x sAA | *r* = -.11, *p* = .552 | *r* = .15, *p* = .368 | *r* = -.20, *p* = .270 | *r* = .05, *p* = .756 |
| Cortisol x sAA | *r* = .27, *p* = .092 | *r* = .18, *p* = .268 | *r* = -.27, *p* = .113 | *r* = .22, *p* = .191 |
| **Total release** | **T1** | **T2** | **T3** | **T4** |
| Cortisol x sAA | *r* = .22, *p* = .191 | *r* = .27, *p* = .113 | *r* = -.14, *p* = .477 | *r* = .24 *p* = .168 |

*Notes*. T1-T4 = four exposure sessions within 2.5 weeks.
